# Occurrence of aflatoxin in agricultural produce from local markets in Burundi and Eastern Democratic Republic of Congo

**DOI:** 10.1002/fsn3.787

**Published:** 2018-10-04

**Authors:** Patchimaporn Udomkun, Charity Mutegi, Tesfamicheal Wossen, Joseph Atehnkeng, Nsharwasi Léon Nabahungu, Emmanuel Njukwe, Bernard Vanlauwe, Ranajit Bandyopadhyay

**Affiliations:** ^1^ International Institute of Tropical Agriculture (IITA) Bujumbura Burundi; ^2^ IITA Nairobi Kenya; ^3^ IITA Lilongwe Malawi; ^4^ IITA Bukavu the Democratic Republic of Congo; ^5^ IITA Ibadan Nigeria

**Keywords:** aflatoxins, Central Africa, crops, fungi, milk and dairy products

## Abstract

Aflatoxins are noxious secondary metabolites, of certain fungal species, found in food and feed. Contamination of a commodity with aflatoxins is associated with production and storage losses, and subsequently less food availability. Aflatoxins can also pose human health risks and represent a barrier to the development of trade, in both domestic and international markets. In this study, samples of cassava, maize, groundnut, beans, soybean, sorghum and milk, and their processed products were collected from local markets in Burundi and Eastern DRC. In order to investigate the levels of aflatoxin, crop samples were analyzed using a single step lateral flow immunochromatographic assay (Reveal Q+), while enzyme‐linked immune‐sorbent assay (ELISA) was used to analyze aflatoxin‐M_1_ in milk, yogurt, and cheese samples. The results revealed the presence of aflatoxins in all samples from both countries, with levels ranging from 1.3 to 2,410 μg/kg. Samples collected from Burundi contained relatively higher (*p* > 0.0.5) levels of aflatoxins. In 51% of all the crops samples, total aflatoxin contamination was above the EU maximum tolerable level of 4 μg/kg. Processed products, particularly from groundnut, maize, and sorghum, had the highest levels of aflatoxin contamination when compared to unprocessed grain. With regard to milk and dairy products, the level of aflatoxin‐M_1_ ranged from 4.8 to 261.1 ng/kg. Approximately 29% of milk and yogurt samples had aflatoxin‐M_1_ higher than the EU regulatory limit of 50 ng/kg, whereas 20% of cheese samples were found to be contaminated at levels higher than the maximum limit of 250 ng/kg. These results can serve as the basis for pre‐ and postharvest approaches to reduce aflatoxin contamination in agricultural commodities in Burundi and Eastern DRC in order to reduce health risk, avoid reduced production in livestock, and open up export markets.

## 1 INTRODUCTION

Nutritional security is effectively achieved when all people at all times consume food of sufficient quantity and quality, in terms of variety, diversity, nutrient content, and safety, to meet their dietary needs and food preferences for an active and a healthy life (FAO/AGN 2012). Contaminated food is one of the major causes of undernutrition, morbidity, and mortality in sub‐Saharan Africa, particularly among children, who are more vulnerable to diseases (Paudyal et al., 2017). Ensuring food safety through the reduction of aflatoxin contamination can contribute significantly to alleviating poverty, increasing food security, and improving nutrition. Also, this has likely positive impacts on enhancing farm productivity, conserving natural resources, as well as improving economic growth by meeting standards in domestic, regional, and international trade.

Among the various mycotoxins, aflatoxins have garnered significant attention due to their negative, and carcinogenic, effects on human and animal health (Klingelhöfer et al., 2018). Although aflatoxins are produced by several *Aspergillus* species, the major causal agent of contamination globally is *A. flavus* (Klich, 2007). There are four major aflatoxins, including B_1_, B_2_, G_1_, and G_2_; however, aflatoxin‐B_1_ is the most toxic and prevalent and is classified as a Group 1A carcinogen by the International Agency for Research on Cancer (IARC 2002). High‐dose exposure to aflatoxins concentrations can cause acute health effects such as vomiting, abdominal pain, and even possible death (Probst, Njapau, & Cotty, 2007; Sherif, Salama, & Abdel‐Wahhab, 2009), while sublethal chronic exposure may lead to liver cancer, stunting in children, and immune system suppression (Chan‐Hon‐Tong, Charles, Forhan, Heude, & Sirot, 2013; Wu & Khlangwiset, 2010). In 1981, for instance, the outbreak of aflatoxicosis as a result of ingestion of maize contaminated with 3.2–12 mg/kg of aflatoxin‐B_1_ caused fatalities in Kenya (Obura, 2013). In another severe aflatoxicosis outbreak, Azziz‐Baumgartner et al. (2005) also reported that aflatoxin contamination was found to be the cause of over 125 deaths during 2004–2005 in Eastern province of Kenya. Williams et al. (2004) estimated that over 5 billion people living in low‐income countries are at risk of chronic exposure to aflatoxins.

The incidence of aflatoxin contamination in major food crops such as maize, groundnut, sorghum, tree nuts, and dried fruits and spices as well as milk and meat products is widespread in warm climates (CAST 2003; Chala et al. 2014; Mutegi, Ngugi, Hendriks, & Jones, 2009; Perrone et al., 2014; Williams et al., 2004). In animals, aflatoxins may lower resistance to diseases, interrupt vaccine‐induced immunity, and adversely affect growth and reproduction, causing serious economic losses (CAST 2003; Fink‐Gremmels, 1999). When animal feeds are infected with aflatoxin‐producing fungi, aflatoxins are introduced into animal source food chains and can be converted to M‐type aflatoxins (De Ruyck, De Boevre, Huybrechts, & De Saeger, 2015; Iqbal, Jinap, Pirouz, & Ahmad Faizal, 2015). Infection and production of aflatoxins by ubiquitous, air‐borne, and soil‐inhabiting species of fungi begin at preharvest stages and may continue to increase until the grain is consumed (Waliyar, Ntare, Diallo, Kodio, & Diarra, 2007; Waliyar et al., 2015).

The interplay between the safety of food and the adequacy of food is therefore crucial when addressing the aflatoxins problem in low‐income countries. An earlier study by Brudzynski, Van Pee, and Kornazewski (1977), for example, showed the presence of aflatoxins up to 1,000 μg/kg in maize and groundnut from the DRC. Recently, Kamika and Takoy (2011) reported that 95% of groundnut samples collected during the dry and the rainy seasons in Kinshasa contained aflatoxin‐B_1_ over the maximum limit of 2 μg/kg prescribed by the EU as the standard for direct human consumption. These studies were, however, limited to maize and groundnut conducted only in the DRC. To expand insight, we conducted a comprehensive investigation on the incidence of aflatoxin contamination in raw and processed materials from cassava, maize, sorghum, beans, soybean, groundnut, and milk in the local markets of Burundi and Eastern DRC.

## 2 MATERIALS AND METHODS

### 2.1 Sample collection

A total of 244 food samples intended for human consumption, including cassava, maize, sorghum, beans, soybean, groundnut, milk, and their products, were randomly collected from local markets in Burundi and Eastern DRC during May–July in 2016. During the time of survey, an average temperature and relative humidity in Burundi were 24.6°C and 70.7%, respectively, while Eastern DRC had an average temperature of 20.5°C and relative humidity of 73.6%. To collect a representative data set, we first obtained the list of villages in each province/district from Provincial Inspection Office. From each province/district, the villages were then randomly selected. In Burundi, the samples were collected from five villages in Gitega province (Gishora, Ruhanza, Tuba‐Murayi, Kobero, and Nyamuhogoti) and eleven villages in Cibitoke province (Rukana, Munyika, Mparambo I, Mparambo II, Rusigabangazi, Kanazi, Murambi, Gasenyi rural, Ruhagarika, Kansege, and Kaburantwa). The samples from the DRC were collected from five villages in Kabare district (Murhesa, Mudaka, Miti Centre, Kavumu, and Katana), Bukavu town and five villages in Uvira district (Kamanyola centre, Katogota, Luberizi, Sange, and Bwegera) of Eastern DRC. These areas were contrasted by altitude, average temperature, and rainfall. Gitega province of Burundi and Kabare district of Eastern DRC is considered as high altitude area, while Chibitoke province of Burundi and Uvira district of Eastern DRC represents low altitude area. The farm households in these areas are mainly subsistence farmers and grow rainfed crops. The main food crops in Gitega province and Kabare district include cereal crops such as maize and legumes, while in Chibitoke province and Uvira district the main crops are cassava and legumes.

For each sample, 1 kg of the commodity was bought and collected from different parts of the seller's container and thoroughly mixed, while 1 L of milk and yoghurt was purchased as sellers prepared in plastic bottle. The sellers were randomly selected from each market, and the type of samples was collected depending on available samples from each seller. After collection, samples were labeled with the name of village and collection date and then subdivided into three portions. The first portion was kept as a backup, while the second was directly used for moisture analysis. The third was examined to determine the level of aflatoxin contamination. All samples were sealed in polyethylene plastic bags under normal atmospheric conditions, whereas the milk and dairy products were kept in plastic bottles. The package seal was carefully inspected to avoid any possibility of leakage. Subsequently, the sealed packages were stored at a temperature of 4°C for dried samples and −4°C for milk and dairy products about 2 weeks, without direct sunlight until further analysis.

### 2.2 Chemical analysis

#### 2.2.1 Moisture content

Moisture content (MC) of each sample was determined by drying the samples in the hot air oven at 105°C for 12 hr, following technique 950.46 (AOAC 2006). The tests were conducted in triplicates, and the moisture content was calculated using the following formula: [(original weight of sample – weight of sample after drying)/original weight of sample] *100.

#### 2.2.2 Analysis of aflatoxins in grains

For each sample except flour, 200 g was ground into fine powder using a laboratory blender (model 37BL85; Dynamics Corporation of America, USA). Approximately 10 g ground sample was added to 50 ml 65% ethanol (v/v) in a 100 ml media bottle. The resulting suspension was shaken (model HS 501 D Shaker; IKA, Germany) at 200 rpm for 3 min to extract aflatoxins. The suspension was allowed to settle, filtered through Whatman No. 1 paper, and filtrate collected.

To analyze the aflatoxins concentration, a Reveal Q+ test kit (Neogen Corporation, USA) was used as a single step lateral flow immunochromatographic assay based on a competitive immunoassay format. A total of 500 μl diluent was mixed with 100 μl of the sample filtrate and then carefully mixed by pipetting up and down five times in a dilution cup. A 100 μl portion of the mixture was transferred to a new clear sample cup. Subsequently, a Reveal Q+ for aflatoxin test strip was placed into the sample cup for 6 min; the strip was removed and inserted to the AccuScan^®^ reader (AccuScan Pro, model AX‐2; Neogen Corporation, Australia). Aflatoxin concentration was displayed in parts per billion (ppb). All samples were analyzed in duplicate from a separate 10 g measure.

#### 2.2.3 Analysis of aflatoxins in milk and dairy products

To determine aflatoxin‐M_1_ in milk and yogurt, the method developed by Gizachew, Szonyi, Tegegne, Hanson, and Grace (2016) was adapted, while the method of Škrbić, Antić, and Živančev (2015) was modified to determine aflatoxin‐M_1_ in cheese products. One hundred milliliter of milk and yogurt samples was warmed to 37°C in a water bath and then centrifuged at 10°C with 3500 *g* for 10 min (model TDL‐5‐A; Lab companion, Korea). After discarding the upper cream layer, the remaining skimmed milk was filtered through Whatman No. 4 filter paper before aflatoxin‐M_1_ analysis. For the cheese products, 2 g of homogenized samples were weighed and blended with 40 ml dichloromethane for 15 min. The filtrates were evaporated via rotary evaporator (model R‐II; Büchi, Postfach, Switzerland) at 60°C. A solution of 0.5 ml methanol, 0.5 ml phosphate buffer saline (PBS), and 1 ml hexane was added to the residue and then centrifuged at 15°C with 2,700 g for 15 min. The lower methanolic phase was collected. Prior aflatoxin‐M_1_ determination, 100 μl of this methanolic phase was diluted with PBS to achieve a dilution of 1:5.

Assay procedure was followed according to the protocol provided by RIDASCREEBN^®^ Aflatoxin‐M_1_ (R‐Biopharm AG, Darmstadt, Germany). Briefly, 100 μl solutions from the mixing wells were transferred to the assay wells coated with aflatoxin‐M_1_ antibodies and incubated for 15 min at room temperature in the dark. After adding 100 μl horseradish peroxidase as a conjugate to aflatoxin‐M_1_, incubation continued for further 30 min. Then the liquid was poured out of the wells, and the wells were washed with PBS‐Tween 20 buffer solution three times. The wells were tapped face down on a layer of absorbent to remove the residual wash buffer. Subsequently, 100 μl of tetramethylbenzidine (TMB), as enzyme substrate, was added into each well, incubated for 15 min at room temperature in the dark, and then 100 μl of the stop solution was added to the microplate wells, which changes the color from blue to yellow. The optical density (OD) was measured at 450 nm using the enzyme‐linked immune‐sorbent assay (ELISA) plate reader (model BDSL, Immunoscan plus; Lab systems, Finland). All analyses were run in duplicates.

Two sets of standard solutions were prepared for aflatoxin‐M_1_ calibration curves. The lower concentrations were in the range of 0.05–0.1 mg/L, whereas the higher concentrations were in the range of 0.1–2.0 mg/L. Samples that were beyond the range of the highest standard concentration were diluted, and the ELISA experiments were repeated.

#### 2.2.4 Total aflatoxin and aflatoxin‐M_1_ validation

To test the sensitivity of the method, the total aflatoxin standard solution at two different concentrations was added to the all samples. The extraction and the recovery of the spiked samples were performed as previously described, in duplicate. The validation of Reveal Q+ and ELISA methods was carried out with the determination of the recoveries and the coefficient of variation (%CV) as presented in Table 1.

**Table 1:** Validation data of methods for total aflatoxins in dried food, milk, and dairy products samples

Category	Total aflatoxin level added (μg/kg)	% Recovery	Coefficient of variation (%CV)
Cassava			
Dried root	2.0	80.2	3.0
	10.0	82.3	3.7
Flour	2.0	85.7	2.9
	10.0	87.6	5.1
*Ubuswage* a	2.0	83.5	6.0
	10.0	81.2	2.9
Bakery products (bread, cookies)	2.0	77.8	4.0
	10.0	83.1	1.2
Maize			
Grain	2.0	93.6	2.1
	10.0	90.8	3.6
Flour	2.0	84.3	4.3
	10.0	90.1	2.4
Sorghum			
Grain	2.0	83.4	1.7
	10.0	85.2	2.5
Flour	2.0	80.5	3.3
	10.0	87.7	4.7
*Germé* b	2.0	78.9	2.6
	10.0	77.6	4.1
Beans			
Grains	2.0	82.8	3.9
	10.0	88.1	1.6
Soybean			
Grains	2.0	83.2	3.2
	10.0	84.6	5.5
Flour	2.0	85.0	4.1
	10.0	88.5	3.6
Groundnut			
Grain	2.0	90.4	1.3
	10.0	92.6	3.2
Roasted	2.0	88.5	2.4
	10.0	83.9	1.8
Flour	2.0	87.2	1.1
	10.0	86.1	2.4
Milk			
Fresh milk	0.5	91.5	1.7
	1.0	101.2	1.1
Yogurt	0.5	95.4	2.8
	1.0	98.5	4.2
Cheese	0.5	91.7	1.9
	1.0	101.5	2.1

a Ubuswage is the traditional cassava product in Central African region.

b Germe is the germinated sorghum for beer processing.

## 3 RESULTS AND DISCUSSION

### 3.1 Moisture content of samples

The MC of grain samples collected from local markets in Burundi and Eastern DRC is shown in Table 2. There was no significant difference in mean MC of the samples from the two countries. In the market, grain samples were mostly kept in open containers, while processed samples were stored in plastic closed containers or paper bag. Only traditionally fermented cassava foods (*ubuswage*) were wrapped in plantain leaves. Overall, the MC ranged between 6.7% and 15.0% for grain samples and between 5.5% and 13.6% for flour, with the lowest being recorded in groundnut flour and the highest in cassava flour. Much higher MC content was recorded in the cassava prepared for *ubuswage*, with an average MC of 59.8%. In addition, the MC of fresh milk, yogurt, and cheese ranged between 79.3% and 89.3%.

**Table 2:** Presence of moisture content in food samples collected from local markets in Burundi and Eastern DRC

Category	Moisture content (g 100/g w.b.)	
	Burundi	Eastern DRC
Cassava		
Dried root	14.95 ± 1.23	–
Flour	13.58 ± 2.41	13.14 ± 1.52
*Ubuswage* a	59.76 ± 4.41	–
Bakery products (bread, cookies)	–	13.07 ± 1.18
Maize		
Grain	11.21 ± 1.49	11.92 ± 1.26
Flour	10.36 ± 1.34	10.58 ± 1.28
Sorghum		
Grain	12.23 ± 0.52	12.52 ± 1.44
Flour	10.76 ± 1.91	10.71 ± 0.76
*Germé* b	10.39 ± 0.65	–
Beans		
Grain	11.52 ± 1.03	11.85 ± 1.30
Soybean		
Dried	9.54 ± 0.30	8.74 ± 1.15
Flour	6.76 ± 2.35	7.60 ± 1.29
Groundnut		
Grain	7.00 ± 1.00	6.65 ± 1.67
Roasted	4.56 ± 1.05	5.21 ± 1.26
Flour	6.52 ± 0.63	5.52 ± 1.04
Milk		
Fresh milk	89.25 ± 1.41	89.12 ± 1.35
Yogurt	88.02 ± 1.25	87.72 ± 1.47
Cheese	–	79.34 ± 1.08

Notes

Value is the mean ± *SD*.

a *Ubuswage* is the traditional cassava product in Central African region.

b *Germe* is the germinated sorghum for beer processing.

### 3.2 Occurrence of aflatoxins in crop samples

The occurrence and concentration of total aflatoxins in crop samples collected from Burundi and Eastern DRC are summarized in Tables 3 and 4. All the 218 samples were contaminated with aflatoxins, which ranged from 1.3 to 2,410.0 μg/kg. Nowadays, the EU has set the strictest standards, such that any products for direct human consumption can only be marketed with concentrations of aflatoxin‐B1 and total aflatoxins not >2 and 4 mg/kg, respectively (EC, 2007, 2010). Likewise, US regulations have specified the maximum acceptable limit for total aflatoxins at 20 mg/kg (Wu, 2006). In India, a tolerance limit of 30 mg/kg for aflatoxins in all foods has been defined. Kenya adopted a maximum allowed level of 10 mg/kg of aflatoxin‐B1 in groundnuts and several grain foods. Brazil has fixed the limit of total aflatoxins in nuts at 30 mg/kg (Freitas‐Silva & Venâncio, 2011). As Burundi and DRC do not have regulations for aflatoxins, in this study, we applied the EU standard as the strictest standards to compare for all crop samples.

**Table 3:** Distribution of total aflatoxins in dried food samples found on local markets in Burundi and Eastern DRC

Category	Burundi				Eastern DRC				Overall			
	Incidence^a^	Average (μg/kg)	Median (μg/kg)	Range (μg/kg)	Incidence^a^	Average (μg/kg)	Median (μg/kg)	Range (μg/kg)	Incidence^a^	Average (μg/kg)	Median(μg/kg)	Range (μg/kg)
Cassava												
Dried root	8/8	3.7	3.6	2.5–5.4	–	–	–	–	8/8	3.7	3.6	2.5–5.4
Flour	10/10	2.8	2.6	1.9–4.6	18/18	2.7	2.7	1.3–5.0	28/28	2.7	2.6	1.3–5.0
*Ubuswage* b	2/2	3.8	4.0	3.3–4.0	–	–	–	–	2/2	3.8	4.0	3.3–4.0
Bakery products (bread, cookies)	–	–	–	–	3/3	3.4	3.3	2.3–5.6	3/3	3.4	3.3	2.3–5.6
Maize												
Grain	10/10	38.7	4.3	2.7–330.0	9/9	10.7	3.2	2.2–73.2	19/19	25.5	3.9	2.2–330.0
Flour	10/10	41.9	5.9	3.2–350.0	9/9	47.9	7.5	2.5–320.0	19/19	44.7	6.9	2.5–350.0
Sorghum												
Grain	12/12	7.1	6.4	5.6–490.0	11/11	4.1	4.3	2.5–5.5	23/23	23.3	4.8	2.5–490.0
Flour	5/5	6.1	5.9	4.0–8.5	7/7	4.9	4.9	3.1–6.5	12/12	5.4	5.2	3.1–8.5
*Germé* c	3/3	6.2	6.3	5.2–6.9	–	–	–	–	3/3	6.2	6.3	5.2–6.9
Beans												
Grains	21/21	3.9	3.7	2.5–6.6	10/10	3.5	3.4	1.9–6.4	31/31	3.7	3.7	1.9–6.6
Soybean												
Grains	8/8	3.4	3.5	2.3–4.1	3/3	3.7	3.8	2.8–4.2	11/11	3.5	3.6	2.3–4.2
Flour	5/5	6.9	4.8	3.5–12.3	4/4	4.1	4.4	2.3–5.5	9/9	5.6	5.7	2.3–12.3
Groundnut												
Grain	7/7	7.1	4.6	3.9–29.3	9/9	3.4	3.4	2.2–5.4	16/16	5.0	3.9	2.2–29.3
Roasted	10/10	220.3	34.0	4.3–1,080.0	11/11	4.0	3.9	2.9–5.7	21/21	107.0	5.3	2.9–1,080.0
Flour	10/10	824.0	550.0	310.0–2,410.0	2/2	1027.5	1010.0	470.0–1,620.0	12/12	857.9	550.0	310.0–2,410.0
Total	121/121	99.6	4.5	1.9–2,410.0	97/97	29.3	3.7	1.3–1,620.0	218/218	68.1	4.0	1.3–2,410.0

a Incidence number is represented by the number of samples with aflatoxins above the detectable level/total sample in a particular category.

b *Ubuswage* is the traditional cassava product in Central African region.

c *Germe* is the germinated sorghum for beer processing.

**Table 4:** Level^a^ of total aflatoxins contamination in dried foods marketed in Burundi and Eastern DRC

Category	Burundi			Eastern DRC			Overall		
	<4 μg/kg	4–10 μg/kg	>10 μg/kg	<4 μg/kg	4–10 μg/kg	>10 μg/kg	<4 μg/kg	4–10 μg/kg	>10 μg/kg
Cassava									
Dried root	7 (87.5)^b^	1 (12.5)	0	–	–	–	7 (87.5)	1 (12.5)	0
Flour	9 (90)	1 (10)	0	16 (88.9)	2 (11.1)	0	25 (89.3)	3 (10.7)	0
*Ubuswage* c	1 (50)	1 (50)	0	–	–	–	1 (50)	1 (50)	0
Bakery products (bread, cookies)	–	–	–	3 (100)	0	0	3 (100)	0	0
Maize									
Grain	4 (40)	4 (40)	2 (20)	7 (77.8)	1 (11.1)	1 (11.1)	11 (57.9)	5 (26.3)	3 (15.8)
Flour	3 (30)	2 (20)	5 (50)	2 (22.2)	3 (33.3)	4 (44.5)	5 (26.3)	5 (26.3)	9 (47.4)
Sorghum									
Grain	0	9 (75)	3 (25)	4 (36.4)	7 (63.6)	0	4 (17.4)	16 (69.6)	3 (13)
Flour	0	5 (100)	0	2 (25.0)	6 (75.0)	0	2 (15.4)	11 (84.6)	0
*Germé* d	0	3 (100)	0	–	–	–	0	3 (100)	0
Beans									
Grain	14 (66.7)	7 (33.3)	0	8 (80)	2 (20)	0	22 (71)	9 (29)	0
Soybean									
Grain	7 (87.5)	1 (12.5)	0	2 (66.7)	1 (33.3)	0	9 (81.8)	2 (18.2)	0
Flour	1 (20)	3 (60)	1 (20)	2 (50)	2 (50)	0	3 (33.3)	5 (55.6)	1 (11.1)
Groundnut									
Dried	1 (14.3)	5 (71.4)	1 (14.3)	7 (77.8)	2 (22.2)	0	8 (50.0)	7 (43.8)	1 (6.2)
Roasted	0	3 (30)	7 (70)	7 (63.6)	4 (36.4)	0	7 (33.3)	7 (33.3)	7 (33.3)
Flour	0	0	10 (100)	0	0	2 (100)	0	0	12 (100)
Total	47 (38.8)	45 (37.2)	29 (24.0)	60 (61.9)	30 (30.9)	7 (7.2)	107 (49.1)	75 (34.4)	36 (16.5)

a The EU permissible level and the WHO advisory level for total AFs are 4 and 10 μg/kg, respectively, for foods intended for direct human consumption.

b The first integer is the number, and the integer in parenthesis is the percent of samples containing a specified level of aflatoxins.

c *Ubuswage* is the traditional cassava product in Central African region.

d *Germe* is the germinated sorghum for beer processing.

About 60% of these samples contained aflatoxins above the EU maximum permissible limit (4 μg/kg) for total aflatoxins in maize intended for human consumption (EC 2007; EC 2010). As other countries found within the tropics, aflatoxin contamination in food commodities from Burundi and Eastern DRC can be attributed to high temperatures and drought conditions driven by climate change, resulting in crop stress which favors *A. flavus* infection in the production field and proliferation during postharvest period (Bandyopadhyay et al., 2016; Kamika, Koto‐te‐Nyiwa, & Tekere, 2016; Kamika & Takoy, 2011; Paterson & Lima, 2010; Schmidt‐Heydt, Abdel‐Hadi, Magan, & Geisen, 2009). In addition, high aflatoxin contamination levels can be compounded by other farm practice factors, including poor weeding, infertile soils particularly in Burundi, poor crop rotation, high planting densities, and delayed time of harvesting. The poor storage of agricultural produce can also lead to accelerated aflatoxin contamination as a result of proliferation of aflatoxin‐producing fungi. This has been demonstrated by many authors (Azziz‐Baumgartner et al., 2005; Mwalwayo & Thole, 2016). Some socioeconomic factors may also contribute to aflatoxins contamination, including informal marketing systems, inadequate transportation modes, unavailability of needed materials, tools, and equipment, lack of information and knowledge on appropriate pre‐ and postharvest managements, and poor governmental regulations and legislations. Moreover, some of these countries have experienced conflicts, resulting in poor outcomes in health, education, and living standards. Food insecurity and malnutrition, especially among children, in resource‐poor households are also common occurrence. It is therefore not surprising that aflatoxin contamination was detected in all of the samples collected in this study, especially in processed products which have lower MC. The average aflatoxin contamination was high in the samples from Burundi (99.6 μg/kg) when compared to those of Eastern DRC (29.3 μg/kg). This result can be explained by the fact that Burundi is relatively hotter and drier, a situation that favors the growth of mycotoxin‐producing fungi. Further details of the incidence of aflatoxin contamination in specific crops are also presented below.

#### 3.2.1 Cassava

Aflatoxin levels in cassava samples ranged from 1.3 to 5.6 μg/kg. More than 88% of the samples met the EU regulatory threshold for aflatoxin of 4.0 μg/kg. All the samples met the proposed East Africa regulatory threshold of 10 μg/kg. Similar observations regarding the low contamination by aflatoxins in cassava are reported in Ghana (Wareing, Westby, Gibbs, Allotey, & Halm, 2001), Republic of Benin (Adjovi et al., 2014; Gnonlonfin et al., 2012) and Tanzania (Sulyok et al., 2015) The occurrence of aflatoxins in cassava chips from Cameroon was only detected after 4 weeks’ storage (Essono et al., 2009). These results suggested that fresh cassava is safe regarding aflatoxin contamination; however, processing methods such as heat treatment, sun drying, or freezing may alter the ability of cassava to block toxinogenesis, leading to secondary contamination. Another possible explanation associated with this observation is that the effect of fermentation process generally employed in the processing of cassava into dried cassava, cassava flour, and *ubuswage* favors the growth of lactic acid bacteria (LAB) or some microorganisms like *Saccharomyces cerevisiae* strains. The ability of these microorganisms to bind or degrade aflatoxins, especially aflatoxin‐B_1_ and aflatoxin‐M_1_, in foods and feeds has been reported (Ahlberg, Joutsjoki, & Korhonen, 2015; El‐Nezami & Gratz, 2011; Peltonen, El‐Nezami, Haskard, Ahokas, & Salminen, 2001). Aflatoxin binding seems to be strongly related to several factors such as LAB strains, matrix, temperature, pH, and incubation time (El‐Nezami & Gratz, 2011; Shetty, Hald, & Jespersen, 2007). Moreover, the MC of cassava has been shown to influence the shelf life of samples rather than aflatoxin occurrence.

#### 3.2.2 Maize

Among the grain samples, the high concentrations of total aflatoxins were obviously detected in maize, followed by groundnut, sorghum, beans, and soybean, respectively (Table 3). Notably, aflatoxin levels in maize flour ranged from 2.5 to 350.0 μg/kg. Kamika et al. (2016) also reported that aflatoxin contamination in the DRC along the maize supply chain. They showed that contamination increased of up to 500 times from preharvest (3.1–103.9 μg/kg) to city stores (2,070.5 μg/kg) and to distribution markets (2,806.5 μg/kg). They attributed this trend to inappropriate storage practices as well as a lack of drying facilities in the country. Similar studies in SSA countries have reported high levels of aflatoxin contamination in maize. Kaaya and Kyamuhangire (2006), for instance, reported more than 20 μg/kg of aflatoxins in maize kernels from Uganda after 6 months of storage, while very high content of aflatoxins in homegrown maize was found in Kenya when compared to purchased or relief maize (Daniel et al., 2011). Lewis et al. (2005) indicated that the contaminated homegrown maize may represent a source of aflatoxin contamination in market maize, especially when local farmers sold a portion of their farm household stores to market vendors. In Tanzania, Kamala et al. (2015) also reported that 87% of maize samples were co‐contaminated with aflatoxins and fumonisins.

#### 3.2.3 Sorghum

In this study, all sorghum samples in grain, flour, and *germé* forms contained detectable concentrations of aflatoxins, ranging between 2.5 and 490.0 μg/kg. Additionally, total aflatoxins exceeded the regulatory levels for direct human consumption as set by the EU in 84.6% of the sorghum samples. The levels of aflatoxin contaminations may also be associated with the poor pre‐ and postharvest practices as well as processing methods. Sorghum, in particular, is used as a malted grain *(germé)* in beer production in Burundi. The traditional processing technique, which involves the use of *Enterobacteruaceae* and molds, may cause aflatoxin contamination in *germé* (Bationo et al., 2015). Although zearalenone is reported as the most common mycotoxin found in sorghum (Chala et al., 2014), high levels of aflatoxins, ranging 340–476 μg/kg, were also found in malted sorghum (Matumba, Monjerezi, Khonga, & Lakudzala, 2011). Another study by Ayalew, Fehmann, Lepschy, Beck, and Abate (2006) reported that about 6% of field samples of sorghum in Ethiopia are contaminated with aflatoxin‐B1 up to 26 μg/kg, whereas Bandyopadhyay, Kumar, and Leslie (2007) found that 5% of sorghum grain samples exceeded the Nigerian safety threshold of 20 μg/kg.

#### 3.2.4 Beans

Aflatoxin was present in 100% of bean samples from Burundi and Eastern DRC and ranged from 1.9 to 6.6 μg/kg. This low level of aflatoxin contamination in the bean samples is perhaps due to the ability of phenolic compounds, particularly gallic and chlorogenic acids, to inhibit fungal amylase activities (Telles, Kupski, & Furlong, 2017). Pagnussatt, Bretanha, Sílvia, Garda‐Buffon, and Badiale‐Furlong (2013) also mentioned that the synergistic effect of different compounds in beans can contribute to a defense barrier against development of toxigenic species. Literature reports a few instances of aflatoxins in red kidney beans, split peas, chickpea, and cowpea such as in Pakistan (Lutfullah & Hussain, 2012).

#### 3.2.5 Soybean

All soybean samples analyzed were positive for total aflatoxins with 40.0% of these samples exceeding 4 μg/kg. The highest concentration of aflatoxins was found in flour than in dried grains. It has been reported that aflatoxin contaminations in soybean are relatively low, but there are conflicting explanations to the possible cause of low aflatoxin contamination in soybean. One of the initial studies associated this phenomenon to the zinc binding ability of phytate in soybean, as it is an important intermediate substrate of aflatoxin biosynthesis (Gupta & Venkitasubramanian, 1975). However, Ehrlich and Ciegler (1985) showed that phytate level does not influence aflatoxin biosynthesis. Burow, Nesbitt, Dunlap, and Keller (1997) hypothesized that lipoxygenase in soybean can produce hydroxyl fatty acids which are capable of inhibiting aflatoxin production in *A. parasiticus*. With regard to aflatoxin inhibition, Mellon and Cotty (2002) reported that soybean grains with lipoxygenase might not deter increased seed pathogen susceptibility, but seed coat integrity and seed viability may play more determinant role in seed resistance to aflatoxin contamination. There is hence the need for further understanding of the possible cause of low aflatoxin contamination in soybean.

#### 3.2.6 Groundnut

In this study, total aflatoxins concentration in groundnut products from the local markets in Burundi and Eastern DRC ranged from 2.2 to 2,410.0 μg/kg. The highest contamination level was found in groundnut flour (2,410 μg/kg), followed with roasted groundnut (1,080 μg/kg) and dried kernels (29.3 μg/kg), respectively. About 69.4% of the groundnut samples exceeded the EU aflatoxin regulatory limits. None of the groundnut flour samples were fit for human consumption according to any existing regulation globally, with some samples surpassing the EU maximum permissible limit of 4 μg/kg by 600‐fold. Aflatoxins were found more in processed groundnut than in unprocessed dried grains (Tables 2 and 3). Processed groundnut, often prepared from low quality groundnut, can be exposed to a wide range of environmental conditions, such as high temperature and humidity as well as to oxygen and mold, which can trigger further increase in aflatoxin contamination. Nonetheless, other factors including biological, nutritional, and climatic factors can be responsible for aflatoxins contamination, especially in groundnut and maize, some of which are either difficult or impracticable to control. Groundnut is a preferred substrate for aflatoxin‐producing fungi (Bankole, Schollenberger, & Drochner, 2006; Ezekiel et al., 2013; Monyo et al., 2012). The range of aflatoxin contamination in groundnut samples in this study was comparable to those reported from local vendors, markets, and retail shops in Nigeria where aflatoxin‐B_1_ detected in 64.2% of dry roasted groundnut (Bankole, Ogunsanwo, & Eseigbe, 2005). In Kenya, about 87.0% of groundnut were contaminated with <4 μg/kg of aflatoxin‐B_1_, while 7.5% exceeded national regulatory limited of 20 μg/kg (Mutegi et al., 2009). Similarly, 70% of groundnut samples from the DRC were found to contain higher than 5 μg/kg aflatoxins (Kamika & Takoy, 2011). Matumba, Van Poucke, Monjerezi, Ediage, and De Saeger (2015) also revealed that groundnut samples from informal markets in Malawi contained aflatoxins up to 47 times as compared with samples destined as export goods.

### 3.3 Occurrence of aflatoxin‐M_1_ in milk and dairy products

Milk and dairy products are important for growth and development as well as maintenance of good health in humans, especially babies and children. The occurrence of aflatoxin‐M_1_ in milk and its products collected in Burundi and Eastern DRC is presented in Tables 5 and 6. According to the EU regulations, the maximum residue level of aflatoxin‐M_1_ in raw milk and dairy products is 50 ng/L, while this level based on USA regulations was adjusted to 500 ng/kg (Campagnollo et al., 2016; Iqbal et al., 2015; Mulunda & Mike, 2014). Aflatoxin‐M_1_ was detected in all samples collected for this study, with concentrations ranging between 4.8 and 261.1 ng/kg. Among the 13 fresh milk samples analyzed, 4 (30.8%) contained aflatoxin‐M_1_ above the maximum permissible limit of 50 ng/kg, as set by the EU for raw milk, heat‐treated milk, and milk for the manufacture of milk‐based products (EC 2006). Of the eight yogurt samples, only two samples (25%) were contaminated with aflatoxin‐M_1_ above the limit of 50 ng/kg, with the concentration ranging between 4.8 and 63.2 ng/kg. Brackett and Marth (1982) explained that the changes in casein structure due to fermentation process may cause adsorption or occlusion of toxins, including aflatoxin‐M_1_, in the precipitate. Montaseri et al. (2014) also referred to this behavior as the possible reason why LAB is capable of removing aflatoxin‐M_1_ from yogurt. Furthermore, the low concentration of aflatoxin‐M_1_ in yogurt might be associated with processing variables such as pH, formation of organic acids, or other fermented by‐products (Govaris, Roussi, Koidis, & Botsoglou, 2002).

**Table 5:** Incidence and concentration of aflatoxin‐M_1_ in milk and dairy products sampled from local markets in Burundi and Eastern DRC

Category	Burundi				Eastern DRC				Overall			
	Incidence^a^	Average (μg/kg)	Median (μg/kg)	Range (μg/kg)	Incidence^a^	Average (μg/kg)	Median (μg/kg)	Range (μg/kg)	Incidence^a^	Average	Median (μg/kg)	Range (μg/kg)
Milk												
Fresh milk	10/10	31.4	43.6	8.4–82.8	3/3	37.3	37.5	25.6–49.8	13/13	42.6	40.5	8.4–82.8
Yogurt	6/6	32.5	33.5	8.2–63.2	2/3	18.0	16.1	4.8–26.0	8/8	27.7	25.0	4.8–63.2
Cheese	–	–	–	–	5/5	170.0	142.0	18.5–261.1	5/5	170.0	142.0	18.5–261.1
Total	16/16	32.5	39.8	8.2–82.8	10/10	37.3	83.3	4.8–261.1	26/26	56.6	32.5	4.8–261.1

a Incidence number is represented by the number of samples with aflatoxins above the detectable level/total sample in a particular category.

**Table 6:** Number of samples with ≤50 ng/kg or more of aflatoxin‐M_1_ concentration in milk and dairy products marketed in Burundi and Eastern DRC

Category	Burundi		Eastern DRC		Overall	
	≤50 ng/kg	>50 ng/kg	≤50 ng/kg	>50 ng/kg	≤50 ng/kg	>50 ng/kg
Milk						
Fresh milk	6 (60)^a^	4 (40)	3 (100)	0	9 (69.2)	4 (30.8)
Yogurt	4 (66.7)	2 (33.3)	2 (100)	0	6 (75)	2 (25)
Cheese	–	–	1 (20)	4 (80)	1 (20)	4 (80)
Total	10 (62.5)	6 (37.5)	6 (60)	4 (40)	16 (61.5)	10 (38.5)

a Figures in parenthesis indicate proportion (%) of samples in a particular category.

Four out of five (80.0%) cheese samples had concentration of aflatoxin‐M_1_ below the EU maximum limit of 250 ng/kg. The contamination of aflatoxin‐M_1_ in these samples can be attributed to the intake of aflatoxigenic mold contaminated feeds by milk‐producing animals. Variability of aflatoxin‐M_1_ in milk and dairy products is influenced by several factors such as geographical region, seasons, type and quality of feed, feed storage conditions, and processing methods and conditions (Gizachew et al., 2016; Škrbić et al., 2015).

Several studies have reported the occurrence of aflatoxin‐M_1_ in milk and dairy products. Milk samples from urban centers in Kenya contained aflatoxin‐M_1_ up to 6,800 ng/L (Kang'ethe & Lang'a, 2009). In Sudan, 95% of milk was contaminated with aflatoxin‐M_1_ ranging between 220 and 6,800 ng/L (Elzupir & Elhussein, 2010), whereas 6–527 ng/L of aflatoxin‐M_1_ was detected in 15% of cow milk samples from Cameroon (Tchana, Moundipa, & Tchouanguep, 2010). The concentration of aflatoxin‐M_1_ varied between 150 and 170 ng/L in commercial and rural milk in South Africa (Mulunda & Mike, 2014), while 8.0% of milk samples in Ethiopia contained aflatoxin‐M_1_ <5 ng/L (Gizachew et al., 2016). In Iran, Feta cheese samples contained aflatoxin‐M_1_ with concentration ranging from 150 to 2,410 ng/kg (Kamkar, Karim, Aliabadi, & Khaksar, 2008), whereas white cheese was contaminated with 52 to 745 ng/kg of aflatoxin‐M_1_ (Fallah, Jafari, Fallah, & Rahnama, 2009). In Serbia, Tomašević et al. (2015) identified that 56.3% of raw milk, 32.6% of heat‐treated milk, and 37.8% of milk product samples contaminated aflatoxin‐M_1_ above the EU maximum residue permitted amount.

## 4 CONCLUSIONS

This first report on the incidence of aflatoxin contamination in agricultural products from local markets in Burundi and Eastern DRC showed that of the 244 crops, milk, and their processed products sampled, the percentage of aflatoxin positive samples was 100%. In addition, 50.9% of crop, 28.6% of milk and yogurt, and 20.0% of cheese samples had aflatoxin concentrations higher than the regulatory limits set by the EU. The processed samples presented higher aflatoxin contamination when compared to unprocessed samples. Therefore, the presence of aflatoxin in local food products from Burundi and Eastern DRC is a problem in the context of food sufficiency, public health, and economic benefits. Appropriate pre‐ and postharvest management strategies need to be promoted among actors along the food value chains, especially farmers and processors, to achieve significant reduction in aflatoxin contamination in agricultural commodities. This can increase food availability, accessibility, utilization, and stability, as well as economic sustainability in the two countries. At the subsistence farm and processing levels, application of biocontrol tools, in conjunction with other aflatoxin‐management practices such as drying and storage technologies, as well as the proper and effective regulatory standards are required as part of efforts to reduce the risk of aflatoxin contamination. Mitigation measures must, however, be backed up by further insights on the causes of contamination and possible variations in contamination levels across regions as well as crop commodities. Further work, for example, on the microbiology, especially on etiology, on‐farm, and postharvest as well as marketing structures need to be studied further. To further strengthen the county's efforts in abating contamination, risk assessments are proposed in order to establish country regulatory thresholds that the local consumer population can depend on and which can be used to monitor safety across the country. These thresholds can also be used to monitor safety of food commodities across the county's boarders.

## CONFLICT OF INTEREST

The authors have no conflict of interests.

## ETHICAL STATEMENT

This study does not involve any human or animal testing.
